# Anaemia in pregnancy: A survey of prevalence and associated factors in Vhembe District, South Africa

**DOI:** 10.4102/safp.v67i1.6182

**Published:** 2025-10-10

**Authors:** Mulimisi Ramavhuya, Robert Mash

**Affiliations:** 1 Division of Family Medicine and Primary Care, Faculty of Health Sciences, Stellenbosch University, Cape Town, South Africa; 2 Department of Family Medicine, Faculty of Health Sciences, University of Limpopo, Polokwane, South Africa

**Keywords:** anaemia in pregnancy, poor nutrition, antenatal care, public health, food insecurity

## Abstract

**Background:**

Anaemia in pregnancy often reflects inadequate nutrition and is linked to adverse pregnancy outcomes. This study aimed to determine the prevalence of anaemia in pregnancy and the related contributing factors in the Vhembe District.

**Methods:**

A descriptive cross-sectional study was conducted among women attending antenatal services from April to June 2021. A total of 419 pregnant women were sampled, with data gathered from their maternity case files and a short questionnaire on medication use.

**Results:**

The prevalence of anaemia in pregnancy in Vhembe District was 32.2%. Of those with anaemia, 58.7% were mild, 38.4% were moderate and 2.9% were severe. Adherence to prescribed oral supplements was 96.5% for iron and 97.3% for folic acid. Reported stock out for iron and folic acid supplements was 27.2% and 30.5% respectively. The mean age of the sample was 26.7 years (SD6.2) while the median gestational age was 30 weeks (IQR 21 to 38). The median gestational age at booking was 16 weeks (IQR 10 to 21) and median parity was one child (IQR 0 to 2). Majority of pregnant women with anaemia had food insecurity.

**Conclusion:**

The prevalence of anaemia in pregnancy in the Vhembe District represents a moderate public health concern. This study adds to the expanding body of knowledge on maternal health by emphasizing food insecurity as a key contributing factor to anaemia in pregnancy.

**Contribution:**

The findings provide locally relevant data that can inform targeted interventions, including integrated food and nutrition support programs within antenatal care services.

## Introduction

### Background

The United Nations (UN) declared 2016–2025 as a ‘decade of action on nutrition’ with activities to accelerate implementation of policies, programmes and increased investment to eliminate all forms of malnutrition.^1^ The declaration of the decade of action ensures political focus on the ‘UN 2030 Agenda for Sustainable Development Goals (SDG)’, where goals two and three are to end hunger, achieve food security, improve nutrition and ensure healthy lives for all by 2030.^1,2^ Anaemia is used as an indicator of poor health and poor nutrition.^3,4^ Globally, iron deficiency remains the commonest cause of nutritional anaemia.^5,6,7^

The World Health Organization’s (WHO) integrated action plan on the double burden of malnutrition, named ‘double-duty actions for nutrition’, is in line with the UN health-related SDGs, which are aimed at eradicating all forms of malnutrition, including nutrition-related anaemia in pregnancy.^2^ The South African government responded to the health-related SDGs and the ‘double-duty actions for nutrition’ by developing the ‘National Food and Nutrition Security Plan (NFNSP) for South Africa 2018–2023’.^8^

The NFNSP had specific interventions, including ensuring adequate provision of iron and other micro-supplements in pregnancy and lactation, food security for South Africans, and training ward-based outreach teams to educate communities on correct food choices. Seventy per cent of doctors, nurses, pharmacists, and dieticians who worked in the communities were to be capacitated through e-learning to provide appropriate nutrition support and counselling by 2023.^8^ Ninety per cent of pregnant women were supposed to have access to an adequate supply of supplements (iron, folic acid, and calcium) by 2023. The proportion of women on iron and folic acid with haemoglobin (Hb) > 10 g/dL at delivery was used as an output indicator. In 2020 the target for the proportion of women with Hb > 10 g/dL at delivery was 60% of the women, while the target for 2023 was 80%.^8^ All five Limpopo districts, including Vhembe, formed part of the 27 districts in South Africa, which were prioritised for the implementation of NFNSP.^8^

Although the routine prescription of ferrous sulphate and folic acid during pregnancy has strong evidence base, the strategy is not always successful. The absorption of iron is affected by the health status of the pregnant woman’s gastrointestinal tract, the type of diet, as well as the presence of minerals and chemicals in the meal. Ingestion of red meat, fish, chicken, liver or ascorbic acid in food increases the absorption of iron, whereas eggs, milk, coffee, tannin-rich tea and calcium cause a decrease in absorption.^9,10^ Calcium supplements should be swallowed at least 4 h apart from iron tablets.^9^ In South Africa, wheat flour and maize meal are fortified with additional iron.^11,12^ Anaemia prevention may be impacted by a poor supply chain, leading to unavailability of iron and folate medication.^12^ Iron and folic acid supplementation strategies also depend on the adherence of pregnant women to the supplements. Adherence to iron and folic acid supplements may be as low as 40% and this requires improvement in patient education and counselling.^13^

Anaemia occurs when the Hb concentration of a person falls below the lower reference level for the population under study.^14,15^ The WHO’s normal reference Hb level in pregnant women is ≥11 g/dL.^5^ Common causes of iron deficiency anaemia in women of childbearing age include poor diet, haemolysis because of parasites such as malaria, schistosomiasis, intestinal parasites such as hookworm, or bleeding during labour.^5^

The 2016 South African maternity care guideline states that pregnant women with an Hb ≥ 10 g/dL should have a repeat of the Hb test between 28 and 32 weeks and again at 36 weeks of gestation. An Hb measurement using a haemoglobinometer is recommended.^12^ Those below 36 weeks gestation with an Hb between 7.9 g/dL and 9.9 g/dL should have a follow-up Hb measurement after 4 weeks of treatment with ferrous sulphate 200 mg orally three times daily and folic acid 5 mg orally daily.^9^

The guideline recommends oral ferrous sulphate 200 mg once per day and oral folic acid 5 mg once per day for the prevention of anaemia in pregnancy in women with Hb ≥ 10 g/dL.^9^ Full-term pregnant women presenting at primary care facilities with an Hb < 10 g/dL are transferred to hospital for delivery. Pregnant women below 36 weeks of gestation presenting with a booking Hb of 8.0 g/dL–9.9 g/dL are booked for a high-risk antenatal clinic if there is no improvement in Hb level following 4 weeks of treatment with oral ferrous sulphate 200 mg three times per day and oral folic acid 5 mg once per day. Antenatal women with booking Hb < 8.0 g/dL are urgently transferred to a district hospital. A pregnant patient with severe anaemia is one with Hb of between 6.0 g/dL and 7.9 g/dL and symptoms of anaemia or one with Hb < 6.0/dL even if she has no symptoms of anaemia.^9^

Anaemia in pregnancy is categorised by the WHO as a problem of public health significance.^3,4^ Mild anaemia in pregnancy is associated with premature delivery, low birth weight and placental hypertrophy.^3,4,15^ Patients with anaemia have reduced resistance to some bacterial infections.^7^ The condition may be associated with more than 39% of maternal deaths.^16^ The risk of maternal death is higher in patients with severe anaemia compared to those with normal Hb.^17^

Iron deficiency in the third trimester has been linked to prolonged or obstructed labour, antepartum haemorrhage, gestational hypertension, pre-eclampsia, and eclampsia.^18^ The need for emergency caesarean sections because of foetal distress, prolonged labour or failed induction is 45% in people with anaemia compared to 29% in women without anaemia.^18^ A significantly higher proportion of patients with stillbirths, low birth weight, very low birth weight, small for gestational age, and preterm delivery is found in the anaemic group.^18^

The commonest indications for blood transfusion in low- and medium-income countries are pregnancy-related complications and childhood anaemia.^19^ Children under the age of 5-years-old and women 15–45-years-old account for 67% of all blood transfusions in low- to medium-income countries.^19^ The majority of blood transfusions in Zimbabwe were for pregnancy- and childbirth-related diagnoses.^20^

The WHO maintains a global database on anaemia as part of their Vitamin and Mineral Nutrition Information system (VMNIS).^3,4^ The organisation has set a global nutrition target of a 50% reduction in the prevalence of anaemia, including in pregnancy, by 2025.^4^ The prevalence of anaemia is used to classify countries by level of public health problem.^3^ An acceptable level is the population-specific prevalence of anaemia of ≤ 4.9%. Countries with a prevalence ≥40% have a severe public health problem. Those with a prevalence of 20.0% – 39.9% have a moderate public health problem, while a prevalence between 5.0% and 19.9% is in the category of a mild public health problem.^3^ Over 80% of the WHO member states have a prevalence of anaemia in pregnancy of more than 20%.^3,4,15^ Of these, 35% have a severe public health problem, while 47% have a moderate public health problem.^3^

Globally, anaemia affects 32.4 million pregnant women, with a prevalence of 38.2%. The prevalence of anaemia in pregnancy is highest in South-East Asia and in Africa at 48.7% and 46.3%, respectively.^3^ Europe and the America regions have the lowest prevalence of anaemia at 24.9% and 25.8%, respectively.^3^ The 24.3% prevalence of anaemia in the Western Pacific region is the lowest, but it is still regarded as a moderate public health problem.^3,4^

The prevalence of anaemia in pregnancy in South Africa (1997–2021) was 29.0%–42.7%.^4^ Most studies have been conducted in KwaZulu-Natal and the prevalence was reported as 58% (in 2003), 40% (in 2006) and 43% (in 2012–14).^21,22,23^ There were no recently published studies on the prevalence of anaemia in pregnancy in Limpopo’s Vhembe district. The proportion of households with inadequate access to food in Limpopo province is 6.4%, against the average for South Africa of 21.3%.^24^

Any efforts towards prevention of anaemia, including anaemia in pregnancy, have major medical, social, and economic advantages.^25^ Interventions to eliminate nutritional anaemia involve collaboration of different sectors, including the Department of Agriculture.^26,27^ Nutrition-related educational brochures and advisory services, for recommended diets to prevent nutritional anaemia in pregnancy, are freely available to professionals and the public.^28^

Knowledge of the prevalence of anaemia in vulnerable groups such as pregnant women is important for planning of appropriate interventions to achieve the set targets and to reduce morbidity and mortality from preventable anaemia.^3,4,15,29^ Given the socio-economic challenges in the Vhembe district and the lack of evidence on the prevalence of anaemia in pregnancy in the area, there was a need to conduct this study. Periodic determination of the prevalence of anaemia in pregnancy in all districts is critical to strengthening efforts directed at prevention and elimination of anaemia in pregnancy and in women of child-bearing age.^8^

The aim was to determine the prevalence and severity of anaemia, and associated factors in pregnant women attending antenatal care (ANC) in Vhembe district, Limpopo, South Africa.

The objectives of the study were to determine the prevalence and severity of anaemia in pregnancy, to identify adherence by pregnant women to prescribed iron and folic acid supplements, as well as to examine the availability of iron and folic acid supplements in Vhembe District Community Health Centres.

## Research methods and design

### Study design

The study was a prospective descriptive cross-sectional survey.

### Setting

Vhembe district is one of the five districts of Limpopo province. It has a predominantly rural population of 1 402 779 persons. The population density is 56.9 persons per km^2^.^30^ About 54.0% of the population is female. The unemployment rate in the district is 15.2%, and 21.0% of adults (20 years and older) have matric, while 16.1% have no schooling. Women-headed families make up 51% of households. About 2.8% of the households are in informal dwellings.^31,32^

The district’s institutional maternal mortality ratio (iMMR) was 79 per 100 000 live births.^33^ The antenatal first visit before 20 weeks rate was 69%, against the South African average of 68%. The percentage of fixed primary health care (PHC) facilities with 90% availability of tracer medicines was 58%, versus 84% for the rest of South Africa.^33^

Professional nurses, with basic midwifery training, provide ANC at primary care facilities. High-risk ANC women are referred to the nearest hospital. Pregnant women in the Vhembe district are tested for Hb, Rhesus factor, syphilis, and HIV on their first antenatal visit.^9^ Those with Hb above 10.0 g/dL are prescribed oral ferrous sulphate 200 mg daily and oral folic acid 5 mg daily. Those with Hb less than 10 g/dL take oral ferrous sulphate 200 mg three times daily and oral folic acid 5 mg daily. A full blood count investigation is performed in patients with Hb < 7.9 g/dL and in those with Hb < 10.0 g/dL who do not improve after 4 weeks of treatment.^9^

Vhembe district has seven hospitals that provide maternal care services.^34^ There are six district hospitals and one regional hospital. During the 2015/2016 financial year, three district hospitals were among the 23 worst-performing district hospitals with the highest number of early neonatal deaths in South Africa.^35^ The district has eight community health centres (CHCs) spread across four local municipalities and attached to different hospitals. Community health centres provide antenatal services for low-risk women and shared care for those classified as high risk.

### Study population

The study population was pregnant women of any age attending ANC at all Vhembe district CHCs. The exclusion criteria included those who declined to participate and sign the consent form, those who were in labour and those who were acutely ill on the day the researcher or his assistant attended the facility.

#### Sample size

The researcher used Epi info 7TM STATCAL to calculate a sample size of 419 pregnant women. The assumptions used were a population size of 29 000, a margin of error of 5%, confidence levels of 95% and an expected frequency of 30%. The sample size was then stratified per CHC according to the ANC headcount.

#### Sampling

There were eight CHCs (Thohoyandou, Bungeni, Mphambo, Makhado, Tshilwavhusiku, William Eddie, Mutale and Tiyane) in the Vhembe District providing maternal care services.

The sample included consecutive selection of pregnant women at antenatal clinics at all CHCs in Vhembe District. As women attended the clinics in a random order, this enabled a representative sample. Pregnant women were not notified in advance about the visit of the researcher or his assistants. Consecutive pregnant women were recruited from each CHC on the days that the researcher and his assistants visited that CHC until the required sample size was achieved.

### Data collection

The researcher and his assistants, who were all midwives, prepared packages for each participant. Each package had a participant information leaflet and consent form, a participant data form, and a Brief Medication Questionnaire form. The participant data form had space for capturing the woman’s age in years, gravidity, parity, current gestational age, gestational age at first ANC visit, booking Hb, TB screen result, HIV status, other medical conditions, and laboratory sticker number. The Brief Medication Questionnaire included questions on education and employment, adherence to medication and any problems with obtaining or taking medication. Food insecurity was measured by a question about reducing the size of meals, skipping meals or not having enough money for food.

The project tools were piloted on 42 pregnant women at a CHC in the Vhembe district, but these data were not included in the study. Data were then collected from 01 April 2021 to 30 June 2021. Pregnant women met with the researcher or assistants after their consultation with the midwife. The researcher and his assistants conducted interviews and assisted participants to complete the questionnaires in a separate building away from where midwives conducted their ANC. The questionnaires were administered in Tshivenda, Tsonga or English and captured the data in REDCap^R^. Information from their maternity case record was used to complete the participant data form.

### Data analysis

Data were checked for errors before importing it to the Statistical Package for Social Sciences (SPSS) version 26. It was analysed by the researcher with assistance from his co-author. Descriptive analysis reported on categorical data using frequencies and percentages. Numerical data used means and standard deviations or medians and interquartile ranges, depending on the distribution of the data.

Inferential analysis investigated the relationship between Hb and mean age, parity, gravidity, HIV status, TB screen, food insecurity, matric pass, degree or diploma qualification, adherence to iron and folic acid, iron pills stockout and folic acid stockout using the independent samples *T*-test, Mann–Whitney *U* test or Chi-square test.

### Ethical considerations

The study was approved by Stellenbosch University’s Health Research Ethics Committee (HREC reference number S20/08/166, project ID 17122). Permission was granted by the Limpopo provincial Department of Health (ref LP_2020_09_059), the Vhembe district health Chief Director; and facility managers of the eight Vhembe district CHCs. Participants’ personal identifiers were not captured or stored in any format.

## Results

A total of 419 pregnant women, with a mean age of 26.7 years (s.d. 6.2), were studied (Table 1). The median gestational age of the participants at booking and on the day of interview was 16 weeks (IQR 10–21) and 30 weeks (IQR 21–38), respectively. Their median parity was one child (IQR 0–2), and their median gravidity was two pregnancies (IQR 1–3). Overall, 57.0% of participants had a matric pass, and 21.9% had higher education qualifications. Forty-six of the participants (10.9%) reported household food insecurity.

**TABLE 1:** Characteristics of the sample (*N* = 419).

Variable	*n*	%
**Community Health Centre (CHC)**		
Bungeni	81	19.3
Makhado	45	10.7
Mphambo	50	11.9
Tiyani	28	6.7
Thohoyandou	90	21.5
Tshilwavhusiku	45	10.7
William Eddie	42	10.0
Mutale	38	9.1
**Age groups (years)**		
≤ 19	51	12.2
20–24	124	29.9
25–29	106	25.3
30–34	85	20.3
≥ 35	53	12.6
**Gravidity**		
1	144	34.4
2–4	251	59.9
≥ 5	24	5.7
**Parity**		
0	151	36.0
1	120	28.6
2–4	114	27.2
≥ 5	3	0.7
Not recorded	31	7.4
**Gestational age at first antenatal visit (weeks)**		
≤ 12	115	27.4
13–20	207	49.4
21–24	56	13.4
≥ 25	38	9.1
Not recorded	3	0.7
**Education**		
Never attended school	3	0.7
Some school but no matric	176	42.0
Passed matric	239	57.0
Not answered	1	0.2
**Higher education (diploma or degree)**		
No	323	77.1
Yes	92	21.9
Not answered	4	0.9
**Participants with food insecurity**		
Yes	46	10.9
No	366	87.3
Not answered	7	1.7

Table 2 presents clinical data on the participants. The prevalence of anaemia was 32.9% (138/419). Of those with anaemia, 58.7% were mild, 38.4% were moderate and 2.9% were severe. The prevalence of HIV infection among the participants was 10.9%, and only 0.5% had a positive TB screen.

**TABLE 2:** Anaemia and clinical data at first antenatal visit (*N* = 419).

Variable	*n*	%
**Haemoglobin (g/dL)**		
≤ 6.9	4	0.9
7.0–9.9	53	12.6
10.0–10.9	81	19.3
≥ 11.0	274	65.4
Not recorded	7	1.7
**HIV status**		
Positive	46	10.9
Negative	361	86.2
Unknown	12	2.8
**TB screen**		
Positive	2	0.5
Negative	280	66.8
Not done	137	32.7

HIV, human immunodeficiency virus; TB, tuberculosis.

Almost all participants had swallowed iron and folate supplements during the past week (Table 3). Poor adherence (taking supplements for three or fewer days) was reported in 3.5% of participants with iron supplements and 2.7% with folate supplements. Most of the participants thought the supplements were useful and experienced no adverse effects. They could read the instructions and open the pill containers easily. There was some evidence that stockouts may be a problem, with 27.2% reporting stockouts of iron tablets and 30.5% stockouts of folate tablets.

**TABLE 3:** Factors affecting adherence to iron and folic acid pills.

Variables	*n*	%
**Ferrous sulphate**		
Swallowed pills within past 7 days (*N* = 368)	352	95.7
Easy to remember to swallow iron pills (*N* = 365)	302	82.7
Iron pills helpful (*N* = 346)	319	92.2
No problems with iron pills (*N* = 369)	286	77.5
Finding it easy to open/close iron pill bag (*N* = 419)	398	94.9
Finding it easy to read the print on iron pill bag (*N* = 419)	401	95.7
Never informed by midwife of iron pills stockout (*N* = 368)	268	72.8
Received iron pills on the day of interview (*N* = 419)	375	89.5
**Folic acid**		
Swallowed pills within the past 7 days (*N* = 368)	348	94.6
Easy to remember to swallow folic pills (*N* = 351)	315	89.7
Folic acid pills helpful (*N* = 339)	317	93.5
No problems with folic acid pills (*N* = 359)	302	84.1
Finding it easy to open/close folic acid pill bag (*N* = 419)	378	90.2
Finding it easy to read the print on folic acid pill bag (*N* = 419)	402	95.9
Never informed by midwife of folic acid pills stockout (*N* = 367)	255	69.5
Received folic acid pills on the day of interview (*N* = 419)	395	94.3
**Iron and folic acid**		
Not bothered by the number of iron and folic acid pills (*N* = 358)	290	81.0
Finding it easy to get iron and folic acid pills at clinic (*N* = 368)	337	91.6

Table 4 compares anaemic and non-anaemic women. The anaemic group was not significantly different in age from the non-anaemic group (mean 25.7 years [s.d. 5.8] versus 27.2 years [s.d. 6.4] and *p* = 0.222). Women with lower parity and gravidity were significantly more likely to be anaemic, as were women from households with food insecurity (Table 4). Tuberculosis screen had no significant effect on the prevalence of anaemia (*p* = 0.072). Women with anaemia took significantly more iron tablets than those without anaemia [median doses per week of 12 doses (95% CI [confidence interval] 12–14) versus 7 doses (95% CI 7–12), *p* = 0.002]. There was no difference between anaemic and non-anaemic women in consumption of folate tablets (95% CI 7–7, *p* = 0.251).

**TABLE 4:** Comparison of anaemic and non-anaemic participants.

Variable	Anaemic		Non-anaemic		*p*-value
	*n*	%	*n*	%	
**Parity (*N* = 381)**	-	-	-	-	0.023
0	59	48.8	89	34.2	-
1	38	31.4	80	30.8	-
2–4	24	19.9	88	33.8	-
> 5	0	0.0	3	1.2	-
**Gravidity (*N* = 412)**	-	-	-	-	0.028
1	61	44.2	80	29.2	-
2–4	71	51.5	176	64.3	-
> 5	6	4.3	18	6.5	-
**HIV status (*N* = 401)**	-	-	-	-	0.480
Positive	18	13.4	27	10.1	-
Negative	116	86.6	239	89.5	-
Unknown	0	0.0	1	0.4	-
**TB screen (*N* = 401)**	-	-	-	-	0.072
Positive	0	0.0	2	0.7	-
Negative	83	61.9	191	71.5	-
Unknown	51	38.1	74	27.7	-
**Food insecurity (*N* = 405)**	-	-	-	-	0.003
Yes	24	17.6	21	7.8	-
No	112	82.4	248	92.2	-
**Matric pass (*N* = 411)**	-	-	-	-	0.291
Yes	83	60.1	151	55.3	-
No	55	39.9	122	44.7	-
**Degree or Diploma (*N* = 408)**	-	-	-	-	0.464
Yes	27	19.7	62	22.9	-
No	110	80.3	209	77.1	-
**Iron pills stockout (*N* = 366)**	-	-	-	-	0.576
Yes	35	27.8	65	27.1	-
No	91	72.2	175	72.9	-
**Folic acid stockout (*N* = 365)**	-	-	-	-	0.215
Yes	44	35.2	68	28.3	-
No	81	64.8	172	71.7	-

HIV, human immunodeficiency virus; TB, tuberculosis.

## Discussion

### Summary of key findings

The prevalence of anaemia in pregnancy at the first ANC visit in Vhembe District was 32.9%. Among the anaemic women, 58.7% had mild anaemia, 38.4% had moderate anaemia and 2.9% had severe anaemia. The mean age of the sample was 26.7-years-old (s.d. 6.2), while the median gestational age was 30 weeks (IQR 21–38). The median gestational age at booking was 16 weeks (IQR 10–21), and the median parity was 1 child (IQR 0–2).

Anaemic women had lower gravidity, lower parity and higher food insecurity than the non-anaemic women. Prevalence of anaemia was not associated with HIV status, TB screen result, education level or the age of the participant. Adherence to the prescribed iron and folic acid tablets was equal in the anaemic and the non-anaemic groups.

Adherence to prescribed oral supplements was 96.5% for iron and 97.3% for folic acid. The reported stockout for iron and folic acid supplements was 27.2% and 30.5%, respectively. The majority of pregnant women with anaemia had food insecurity.

### Discussion of key findings

The prevalence of anaemia in pregnancy of 32.9% in the Vhembe district is in the WHO category of a moderate public health problem (prevalence between 20.0% and 39.5%).^3^ Vhembe is similar to the global average (38.5%) and in the same WHO category as Europe, United States (US) and the West Pacific.^3^ The district is, however, better than Southeast Asia (48.7%) and the rest of the African region (46.3%), which are both in the category of a WHO severe public health problem.^3^

KwaZulu-Natal appears to be one province in South Africa where anaemia in pregnancy was found to be a major public health problem, much worse than in Vhembe and far above the average prevalence in South Africa of 30.0%. The uThungulu district and Addington hospital in KwaZulu-Natal had a prevalence of 57.7% and 42.7%, respectively, both in the category of a WHO severe public health problem.^24,25,26^ Better food security and lower average booking gestational age are probably the reasons for the Vhembe district’s lower prevalence of anaemia compared to the findings in KwaZulu-Natal, where the majority of their participants booked after 20 weeks gestation.^21,23,24,31,33^

In our Vhembe study, lower parity and lower gravidity were associated with anaemia. This contrasts with the findings in the 10 East African countries and the Indian district of Raichur, where nulliparity was a lower risk factor for anaemia and the prevalence of anaemia was high in multigravida women.^30,36,37,38^ Multigravida women in Vhembe booked ANC earlier when compared to those in East Africa and India.^30^

Food insecurity was significantly associated with anaemia in pregnancy. The prevalence of anaemia in communities and countries is positively related to an increase in food insecurity.^3,4^ The commonest cause of anaemia in South African women is nutritional iron deficiency.^39^

The Sixth South African HIV Prevalence, Incidence and Behaviour Survey found that HIV prevalence in Limpopo was relatively low and had dropped from 10.1% in 2017 to 8.9% in 2022.^40^ Nevertheless, the prevalence in this group of pregnant women from Vhembe was lower than expected as numbers of HIV infected women in their reproductive years are usually disproportionately higher.

### Strengths and limitations

The proportion of South African pregnant women who consult at 30 weeks gestation is not known. Our study looked at booking Hb levels before the women received supplements (median gestational age 16 weeks). However, the National Food and Nutrition Plan has Hb at delivery as one of the outcome indicators. Nevertheless, this study shows the degree of anaemia at booking. Adherence to iron and folic acid supplements was based on self-reporting and there may be a social desirability bias. Types of anaemia were not determined, as further haematological laboratory tests were not available. The findings can be generalised to the Vhembe district in Limpopo Province but not to a wider population.

### Recommendations

The prevalence of anaemia was categorised as a moderate public health problem, and this implies that the district needs to address iron and folic acid supply chain issues as well as screen for food insecurity in pregnant women. Supplementary food should be offered to those with food insecurity, and education on food choices should be offered to all pregnant women. Food insecurity should also be tackled in a broader primary health care approach through multisectoral action and community engagement.

## Conclusion

In Vhembe, according to the WHO categorisation, the prevalence of anaemia in pregnancy is a problem of moderate public health significance (32.9% at booking). Among the anaemic women, 58.7% had mild anaemia, 38.4% had moderate anaemia and 2.9% had severe anaemia. Anaemic women had lower gravidity, lower parity, and higher food insecurity than the non-anaemic women. Prevalence of anaemia was not associated with HIV status, TB screening results, education level or age. Adherence to the prescribed iron and folic acid tablets was reportedly high, and there was no difference between anaemic and non-anaemic. The reported stockout for iron and folic acid supplements was 27.2% and 30.5%, respectively. Attention needs to be given to food insecurity, nutrition education, supplements and a reliable supply chain.
